# On the Optimal File Size of Capacity-Achieving Byzantine-Resistant Private Information Retrieval Schemes †

**DOI:** 10.3390/e28010015

**Published:** 2025-12-23

**Authors:** Stanislav Kruglik, Han Mao Kiah, Son Hoang Dau, Huaxiong Wang

**Affiliations:** 1 Department of Electrical and Photonics Engineering, Technical University of Denmark, 2800 Kongens Lyngby, Denmark; 2 School of Physical and Mathematical Sciences, Nanyang Technological University, Singapore 637121, Singapore; hmkiah@ntu.edu.sg (H.M.K.); hxwang@ntu.edu.sg (H.W.); 3 School of Computing Technologies, Royal Melbourne Institute of Technology, Melbourne 3001, Australia; sonhoang.dau@rmit.edu.au

**Keywords:** security and privacy, information retrieval, Byzantine resistance, file size, capacity

## Abstract

We consider the problem of designing a Private Information Retrieval (PIR) scheme for *n* files replicated on *k* servers that can collude and return incorrect answers. Our goal is to correctly retrieve a specific message while keeping its identity private from the database servers. We focus on minimizing download costs and propose PIR schemes with minimal download costs and the smallest file size (proportional to the number of involved servers). Motivated by the possible presence of stragglers, we extend our previous conference results and propose a scheme in which the number of participating servers may vary.

## 1. Introduction

*A Private Information Retrieval (PIR)* scheme is a tool to retrieve a given file $f^{\left(\iota \right)}$ from a database $x=(f^{\left(1\right)},\ldots ,f^{\left(n\right)})$, while keeping its identity ι∈[n] private for the database servers [1,2]. This setup has many related practical applications, including protecting the identity of stock market records reviewed by investment funds and efficiently retrieving blockchain transactions for Ethereum and Bitcoin lightweight clients [3,4]. In this paper, we are interested in multi-server PIR settings, in which each of the *k* servers stores the database, and the client queries each server once. The goal in the classical PIR setting is to keep the identity of the retrieved file private from up to *t* honest-but-curious servers. In the PIR literature, such a scheme is called a *t-private k-server PIR scheme*, and this property is known as *t-privacy*. However, such a setup assumes that servers are honest-but-curious and that they provide correct answers, which cannot be guaranteed in the cloud environment. For example, servers may store outdated databases and, consequently, provide erroneous answers. This case corresponds to the *unsynchronized PIR problem* in the literature [5]. A more severe case arises when some servers are controlled by an adversary who aims to confuse the user by sending corrupted answers. This scenario is known as the *PIR with adversarial servers problem* [6].

Motivated by the challenges mentioned above, in this paper we focus on the scenario in which the user can retrieve the correct file of interest in the presence of up to *b* incorrect responses from servers, known as *b*-Byzantine resistance [7,8,9,10]. We note that errors introduced by Byzantine servers can be intentional or unintentional; however, in both cases, the user must still be able to retrieve the desired message. In what follows, we focus on the case of an omniscient Byzantine adversary with zero probability of error, while leaving the case of a limited-knowledge adversary with a vanishingly small probability of error, as per [11], for further research.

One of the main performance metrics of PIR schemes is the *download rate*, defined as the ratio of the retrieved file size to the total amount of information downloaded by the user. The maximum achievable download rate is known under the name of *capacity* [12]. Maximizing the download rate for PIR schemes in different scenarios is a well-studied scientific problem [1]. The capacity of Byzantine PIR was established in [7], where the authors also proposed a general achievable scheme based on MDS codes. This involves dividing each file into multiple sub-packets, with the number of sub-packets denoted as the *sub-packetization*. In [7], the scheme had a sub-packetization value of ${\left(k-2b\right)}^{n}$, while the problem of determining its minimum value was posed as an open problem by the authors. Since the proposed construction relies on MDS codes, it requires a sufficiently large finite field size. To take this factor into account, in this paper we focus on the total file size. Note that a large number of field elements makes storage and indexing complicated, while a large field size affects the speed of the involved operations [13]. The main result of this paper is the first capacity-achieving Byzantine-resistant PIR scheme with small file size, which fills the existing gap in the research literature and answers the question posed in [7].

There has been considerable research on reducing the file size and finding its minimum value for different PIR setups in the capacity-achieving regime, where the file size is typically large. The latter includes *1-colluding replicated PIR* [14], *1-colluding MDS-coded PIR* [15,16,17], and *t-colluding replicated PIR* [18]. However, to the best of our knowledge, there are no papers that consider a similar problem for Byzantine-resistant PIR. To address this gap, in this paper, we continue our study from [19] and propose a *b*-Byzantine-resistant *k*-server PIR scheme attaining asymptotic capacity and with a small file size.

We begin with a *t*-private PIR scheme based on a recently proposed communication-efficient secret sharing scheme with a small share size from [20]. In this scheme, we represent the whole database as a single vector, and our goal is to privately retrieve certain subsets of its elements. To achieve this, we construct queries such that the desired elements of the vector correspond to the values of a polynomial of a certain degree at specific points. The servers then provide the values of this polynomial at different points, or some function derived from them. In our case, this function is the trace mapping, and we rely on the secret reconstruction algorithms from [20]. To transform this scheme into a Byzantine-resistant setup, inspired by [21], we utilize the idea of constructing a generalized Reed–Solomon code from traces downloaded from servers. The resulting scheme is non-universal, meaning that we have a fixed number of servers with certain restrictions that must participate in the retrieval process. This was described in the conference version of this paper [19]. In this journal version, we generalize the scheme to a universal setup in which there is a finite set of possible values for the number of servers participating in recovery. This makes it possible to configure the scheme based on the current number of available servers. To achieve this, we increase the extension degree of the underlying field and utilize the fact that the extended field can contain several intermediate fields with different extension degrees. Within each of these intermediate fields, the corresponding non-universal scheme can be applied. Since the non-universal scheme is a special case of the universal one, in this paper we present only the universal scheme. We also formally prove that the file size in this scheme is optimal among all capacity-achieving Byzantine-resistant PIR schemes with a sufficiently large number of files.

The rest of this paper is organized as follows: In Section 2, we introduce all of the definitions that we need and list state-of-the-art results. Section 3 is devoted to an explanation of the universal Byzantine-resistant PIR scheme. In Section 4, we derive a lower bound on the file size. Finally, in Section 5, we present a comparison of the proposed scheme with another Byzantine-resistant PIR scheme that attains the capacity for an asymptotically large number of files and allows us to vary the number of participating servers. In Section 6, we provide the conclusions.

## 2. Preliminaries

### 2.1. Notations

For any prime power *q*, we denote the finite field with *q* elements by $F_{q}$ and call it *the base field*. Let $F_{q^{e}}$ be its extension of degree *e*, and call it *the extended field*. An extension of the field $F_{q}$ of degree λ, where λ|e, forms an *intermediate field* $F_{q^{\lambda }}$, which is a subfield of $F_{q^{e}}$. For any $\xi \in F_{q^{e}}$ we define the *trace-mapping function* from $F_{q^{e}}$ to $F_{q^{\lambda }}$ as ${\mathrm{Tr}}_{F_{q^{e}}/F_{q^{\lambda }}}\left(\xi \right)=\sum_{i=0}^{\frac{e}{\lambda }-1}\xi^{{\left(q^{\lambda }\right)}^{i}}$. We note that this is an $F_{q^{\lambda }}$-linear function. It is clear that $F_{q^{e}}$ can be seen as an $F_{q^{\lambda }}$-linear space of dimension e/λ. We can define an $F_{q^{\lambda }}$-basis $\Theta_{1},\ldots ,\Theta_{e/\lambda }$ and its trace-dual basis $\eta_{1},\ldots ,\eta_{e/\lambda }$. It is clear that ${\mathrm{Tr}}_{Fq^{e}/Fq^{\lambda }}\left(\Theta_{i}\eta_{j}\right)=1$ if i=j, and ${\mathrm{Tr}}_{Fq^{e}/Fq^{\lambda }}\left(\Theta_{i}\eta_{j}\right)=0$ otherwise. By $F_{q^{e}}\left[\xi \right]$ we denote the *ring of polynomials* over $F_{q^{e}}$. The minimal polynomial of an element α of the finite field $F_{q^{e}}$ is a nonzero monic polynomial of the smallest degree in $F_{q^{e}}\left[\xi \right]$, such that its value at the point α is zero. We refer the reader to [22] for an extensive introduction to finite field theory. By H(X) we denote the *entropy* of a discrete random variable *X*, and by I(X;Y) we denote the *mutual information* between two discrete random variables *X* and *Y*. A comprehensive overview of information theory can be found in [23]. The main notations utilized in this paper are listed in Table 1.

Throughout this paper, we consider a database with *n* files, each containing *s* elements of the extended field. The parameter *s* is the *sub-packetization*. We represent the database as the vector $x=[f^{\left(1\right)}\ldots f^{\left(n\right)}]$ of length sn. We define the file $f^{\left(\iota \right)}≜\left[x_{1}^{\left(\iota \right)},\ldots ,x_{s}^{\left(\iota \right)}\right]$ and introduce the following database polynomial over $F_{q^{e}}$ with sn indeterminates $z_{1}^{\left(1\right)},\ldots ,z_{s}^{\left(1\right)},\ldots ,z_{s}^{\left(n\right)}$:

(1) $$\begin{array}{cc} F_{x}\left(z_{1}^{\left(1\right)},\ldots ,z_{s}^{\left(1\right)},\ldots ,z_{s}^{\left(n\right)}\right)= & x_{1}^{\left(1\right)}z_{1}^{\left(1\right)}+\ldots +x_{s}^{\left(1\right)}z_{s}^{\left(1\right)}+\ldots +x_{s}^{\left(n\right)}z_{s}^{\left(n\right)}. \end{array}$$

It is clear that $F_{x}\left(e_{i}^{\left(j\right)}\right)=x_{i}^{\left(j\right)}$ for the indicator vector $e_{i}^{\left(j\right)}$ of length sn with all zeros except a single one in the position s(j−1)+i. As a result, to retrieve file ι, we have to obtain elements $F_{x}\left(e_{1}^{\left(\iota \right)}\right),\ldots ,F_{x}\left(e_{s}^{\left(\iota \right)}\right)$. For example, in the case of n=2 and s=2, we have two files of two elements, $f^{\left(1\right)}=\left[x_{1}^{\left(1\right)},x_{2}^{\left(1\right)}\right]$ and $f^{\left(2\right)}=\left[x_{1}^{\left(2\right)},x_{2}^{\left(2\right)}\right]$. The database is $x=[f^{\left(1\right)},f^{\left(2\right)}]$, and the database polynomial has the following form: $F_{x}\left(z_{1}^{\left(1\right)},z_{2}^{\left(1\right)},z_{1}^{\left(2\right)},z_{2}^{\left(2\right)}\right)=x_{1}^{\left(1\right)}z_{1}^{\left(1\right)}+x_{2}^{\left(1\right)}z_{2}^{\left(1\right)}+x_{1}^{\left(2\right)}z_{1}^{\left(2\right)}+x_{2}^{\left(2\right)}z_{2}^{\left(2\right)}$. It is clear that, to recover the file $f^{\left(1\right)}=\left[x_{1}^{\left(1\right)},x_{2}^{\left(1\right)}\right]$, we need to obtain the values of $F_{x}$ at the points (1,0,0,0) and (0,1,0,0).

### 2.2. k-Server PIR Schemes

Let us formally define the *k-server PIR* scheme for the replicated database. The user aims to retrieve the file ι∈[n] by sending queries $q_{1},\ldots ,q_{k}$ to *k* servers. Upon receiving the query $q_{l}$, each server l∈[k] computes the corresponding answer $a_{l}$ and sends it back to the user. In the *Byzantine PIR* setting, there exists an unknown set of up to *b* servers that may provide incorrect answers to queries. With this introduction, we can now formally define *k-server t-private b-Byzantine-resistant PIR*.

**Definition** **1** (*k*-server *t*-private *b*-Byzantine-resistant PIR)**.** *A k-server t-private b-Byzantine-resistant PIR is a scheme that satisfies the following properties:*

*1.*  *(Privacy) The scheme is t-private, i.e., any subset of t or fewer queries does not reveal any information about the identity of the file of interest.*
*2.*  *(Correctness) The scheme is correct and b-Byzantine-resistant, i.e., the user is always able to successfully decode the file of interest from any k queries and corresponding answers even if up to b answers are incorrect. We note that the set of incorrect answers is not known to the user a priori.*

**Remark** **1.** *By setting b=0, this definition is reduced to a t-private k-server PIR scheme.*

The capacity of a single-round *k*-server *t*-private *b*-Byzantine-resistant PIR scheme under the zero probability of incorrect retrieval and omniscient adversary for the case of 2b+t<k is shown in [7] to be equal to

(2) $$C_{n}\left(t,b,k\right)≜\frac{k-2b}{k}\cdot \frac{1-\frac{t}{k-2b}}{1-{\left(\frac{t}{k-2b}\right)}^{n}}.$$

Given the large number of files in real-life systems, we are interested in *asymptotic capacity* values. Therefore, we let n→∞, and for the case of 2b+t<k, we have the following capacity value of a Byzantine PIR scheme:

(3) $$C\left(t,b,k\right)≜\lim_{n\to \infty }C_{n}\left(t,b,k\right)=\frac{k-2b}{k}\cdot \left(1-\frac{t}{k-2b}\right).$$

### 2.3. A Communication-Efficient PIR Scheme

Let us adopt a communication-efficient secret sharing scheme from [20] to obtain a *k*-server *t*-private PIR scheme with an optimal download rate and sub-packetization s≥1. Within the example of Section 2.1, to obtain the file $f^{\left(1\right)}=\left[x_{1}^{\left(1\right)},x_{2}^{\left(1\right)}\right]$, we deploy a ramp secret sharing scheme with threshold *t* for the vectors (1, 1, 0, 0) and (0, 0, 1, 0) as secrets from [24]. The latter is a four-dimensional curve that passes through these two vectors and *t* other random vectors. Each server, as a query, receives the value of this curve at a designated point. As an answer, the server computes the value of the database polynomial at its curve value. These answers are values of a polynomial of degree s+t−1=r−1, and to obtain the file of interest, we need to recover its values at the points corresponding to the secrets. The latter can be performed either by Lagrange interpolation or through the trace-repair framework. For the convenience of the reader, before providing the details of the whole scheme and proving its correctness, let us illustrate the process using $k_{d}=ds+t$ servers and the trace-repair framework. 

At the beginning, for all components i∈[s] of the file, we compute the values of $h_{i1}^{\left(k_{d}\right)}\left(\alpha_{i}\right)=\frac{\eta_{1}^{\left(k_{d}\right)}}{u_{i}\prod_{j\in [s]\setminus \{i\}}{\tilde{f}}_{j}^{\left(k_{d}\right)}\left(\alpha_{i}\right)},\ldots ,h_{id}^{\left(k_{d}\right)}\left(\alpha_{i}\right)=\frac{\eta_{d}^{\left(k_{d}\right)}}{u_{i}\prod_{j\in [s]\setminus \{i\}}{\tilde{f}}_{j}^{\left(k_{d}\right)}\left(\alpha_{i}\right)}$. Here, $\left\{\Theta_{1}^{\left(k_{d}\right)},\ldots ,\Theta_{d}^{\left(k_{d}\right)}\right\}$ is a basis of $F_{q^{e}}$ over $F_{q^{\lambda_{d}}}$, $\left\{\eta_{1}^{\left(k_{d}\right)},\ldots ,\eta_{d}^{\left(k_{d}\right)}\right\}$ is its trace-orthogonal basis, ${\tilde{f}}_{j}^{\left(k_{d}\right)}$ is the minimal polynomial of $\alpha_{j}$ over $F_{q^{\lambda_{d}}}$, and $v_{i}$ and $u_{i}$ are multipliers of the generalized Reed–Solomon code. Without loss of generality, assume that we download the following traces from servers $1,\ldots ,k_{d}$: ${\mathrm{Tr}}_{F_{q^{e}}/F_{q^{\lambda_{d}}}}\left(v_{1}\phi \left(\beta_{1}\right)\right)=a_{1},\ldots ,{\mathrm{Tr}}_{F_{q^{e}}/F_{q^{\lambda_{d}}}}\left(v_{1}\phi \left(\beta_{k_{d}}\right)\right)=a_{k_{d}}$. Each trace is an element of $F_{q^{\lambda_{d}}}$. After this, we find $x_{i}^{\left(\iota \right)}=-\sum_{\delta =1}^{d}\theta_{\delta }^{\left(k_{d}\right)}\left(\sum_{l\in I}h_{i\delta }^{\left(k_{d}\right)}\left(\beta_{l}\right)a_{l}\times \prod_{j\in [s]\setminus \{i\}}{\tilde{f}}_{j}^{\left(k_{d}\right)}\left(\beta_{l}\right)\right)$ and obtain the file $f^{\left(\iota \right)}=\left[x_{1}^{\left(\iota \right)},\ldots ,x_{s}^{\left(\iota \right)}\right]$.

*Scheme $\Pi_{1}$: k*-server *t*-private PIR
    Let *t*, *r*, $k_{d}$, and *k* be positive integers satisfying $t<r\leq k_{d}\leq k\leq q$, s=r−t, $k_{d}\in \Delta =\left\{k_{1},\ldots ,k_{\left|\Delta \right|}\right\},$ where $k_{d}=ds+t\leq k$ and $d\in N^{*}$. Denote by $e≜\frac{\mathrm{lcm}(k_{2}-t,\ldots ,k_{\left|\Delta \right|}-t)}{s}$. Let $\Omega_{\alpha }=\left\{\alpha_{1},\ldots ,\alpha_{s}\right\}\subset F_{q^{e}}$, $\Omega_{\chi }=\left\{\chi_{1},\ldots ,\chi_{t}\right\}\subset F_{q^{e}}$, and $\Omega_{\beta }=\left\{\beta_{1},\ldots ,\beta_{k}\right\}\subseteq F_{q}$ be publicly known non-intersecting sets such that all elements of $\Omega_{\alpha }$ are roots of distinct monic irreducible polynomials of degree *e* over $F_{q}$.
*Query Generation Algorithm:* To retrieve the file ι∈[n], the user randomly generates *t* random vectors $r^{\left(1\right)},\ldots ,r^{\left(t\right)}\in F_{q^{e}}^{sn}$ and draws a random degree-(t+s−1) curve$$\begin{array}{c} g\left(\xi \right)≜\sum_{o=1}^{s}\prod_{p\in [s]\setminus \{o\}}\left(\frac{\xi -\alpha_{p}}{\alpha_{o}-\alpha_{p}}\right)\prod_{p=1}^{t}\left(\frac{\xi -\chi_{p}}{\alpha_{o}-\chi_{p}}\right)e_{o}^{\left(\iota \right)}+\sum_{o=1}^{t}\prod_{p=1}^{s}\left(\frac{\xi -\alpha_{p}}{\chi_{o}-\alpha_{p}}\right)\prod_{p\in [t]\setminus \{o\}}\left(\frac{\xi -\chi_{p}}{\chi_{o}-\chi_{p}}\right)r^{\left(o\right)} \end{array}$$
that resides in $F_{q^{e}}^{sn}$ and passes through points $\left(\alpha_{1},e_{1}^{\left(\iota \right)}\right),\ldots ,\left(\alpha_{s},e_{s}^{\left(\iota \right)}\right)$. The query to server l∈[k] is $g(\beta_{l})$. We note that g depends on the retrieved index ι, but we omit the subscript ι for readability. As introduced earlier, $e_{i}^{\left(\iota \right)}$ is an indicator vector of length sn with all zeros except for a single one at position s(ι−1)+i.*Answer Generation Algorithm:* Upon receiving the query $g(\beta_{l})$, the server l∈[k] computes $F_{x}\left(g\left(\beta_{l}\right)\right)$, where $F_{x}$ is the database polynomial over $F_{q^{e}}$ determined in (1). We can observe that $F_{x}\left(g\left(\xi \right)\right)=\phi \left(\xi \right)$, which is a polynomial in ξ of degree s+t−1=r−1, such that $\phi \left(\alpha_{i}\right)=F_{x}\left(g\left(\alpha_{i}\right)\right)=F_{x}\left(e_{i}^{\left(\iota \right)}\right)=x_{i}^{\left(\iota \right)}$ for i∈[s].
-Each involved server l∈I⊆[k], $\left|I\right|=k_{d}$ responds with$$a_{l}={\mathrm{Tr}}_{F_{q^{e}}/F_{q^{\lambda_{d}}}}\left(v_{l}\phi \left(\beta_{l}\right)\right)\in F_{q^{\lambda_{d}}},$$
where $\lambda_{d}=\frac{es}{k_{d}-t}$ and$$v_{l}=\left\{\begin{array}{cc} 1 & \mathrm{if}\;d=1,\\ \prod_{j=1}^{s}{\left(\beta_{l}-\alpha_{j}\right)}^{-1}\times \prod_{j\in I\setminus \{l\}}{\left(\beta_{l}-\beta_{j}\right)}^{-1} & \mathrm{otherwise}. \end{array}\right.$$Clearly, when d=1, we have ${\mathrm{Tr}}_{F_{q^{e}}/F_{q^{\lambda_{d}}}}\left(x\right)=x$ and, as a result, $a_{l}=\phi \left(\beta_{l}\right)$.*File retrieval algorithm*-For retrieval from answers from $k_{1}=r$ servers, the user applies the Lagrange interpolation formula to recover the polynomial ϕ(ξ) and, as a result, obtains $\phi \left(\alpha_{1}\right),\ldots ,\phi \left(\alpha_{s}\right)$.-For retrieval from answers from servers within the set I⊆[k], $\left|I\right|=k_{d}$, d≠1, the user prepares a basis $\left\{\theta_{1}^{\left(k_{d}\right)},\ldots ,\theta_{d}^{\left(k_{d}\right)}\right\}$ for $F_{q^{e}}$ over $F_{q^{\lambda_{d}}}$ and its trace-orthogonal basis $\left\{\eta_{1}^{\left(k_{d}\right)},\ldots ,\eta_{d}^{\left(k_{d}\right)}\right\}$. Then, for all i∈[s] and δ∈[d], the user chooses polynomials $h_{i\delta }^{\left(k_{d}\right)}\left(\xi \right)\in F_{q^{\lambda_{d}}}\left[\xi \right]$ of degree less than *d* so that$$h_{i\delta }^{\left(k_{d}\right)}\left(\alpha_{i}\right)=\frac{\eta_{\delta }^{\left(k_{d}\right)}}{u_{i}\prod_{j\in [s]\setminus \{i\}}{\tilde{f}}_{j}^{\left(k_{d}\right)}\left(\alpha_{i}\right)},$$
where ${\tilde{f}}_{j}^{\left(k_{d}\right)}\left(\xi \right)$ is the minimal polynomial of $\alpha_{j}$ over $F_{q^{\lambda_{d}}}$, and$$u_{i}=\prod_{j\in [s]\setminus \{i\}}{\left(\alpha_{i}-\alpha_{j}\right)}^{-1}\times \prod_{l\in I}{\left(\alpha_{i}-\beta_{l}\right)}^{-1}.$$The user retrieves the file of interest by$$\begin{array}{c} x_{i}^{\left(\iota \right)}=\phi \left(\alpha_{i}\right)=-\sum_{\delta =1}^{d}\theta_{\delta }^{\left(k_{d}\right)}\left(\sum_{l\in I}h_{i\delta }^{\left(k_{d}\right)}\left(\beta_{l}\right)a_{l}\times \prod_{j\in [s]\setminus \{i\}}{\tilde{f}}_{j}^{\left(k_{d}\right)}\left(\beta_{l}\right)\right), \end{array}$$
for all i∈[s].

**Theorem** **1.** Scheme $\Pi_{1}$ is a k-server t-private PIR over $F_{q^{e}}$ with retrieval threshold $k_{1}=r$ and file size $\mathrm{lcm}\left(k_{2}-t,\ldots ,k_{\left|\Delta \right|}-t\right)\log\left(q\right)$ bits that allows retrieval from any $k_{1},\ldots ,k_{\left|\Delta \right|}$ servers and achieves the asymptotic capacity (3) for b=0 and any given s and r so that $t<r\leq k_{d}\leq k\leq q$, s=r−t, and $k_{d}\in \Delta =\left\{k_{1},\ldots ,k_{\left|\Delta \right|}\right\}$ for $k_{d}=ds+t$, $d\in N^{*}$, and $e=\frac{\mathrm{lcm}(k_{2}-t,\ldots ,k_{\left|\Delta \right|}-t)}{s}$.

**Proof.**  Let us prove privacy and correctness properties according to Definition 1 in case of b=0 and show that responses from *r* servers are enough for file retrieval. The proof is similar to the proof from [20], but for the convenience of the reader, we present it here in full detail.

To prove the privacy, we need to show that $I(g\left(\beta_{l_{1}}\right),\ldots ,g\left(\beta_{l_{t}}\right);e_{1}^{\left(\iota \right)},\ldots ,e_{s}^{\left(\iota \right)})=0$, for any subset $\left\{l_{1},\ldots ,l_{t}\right\}\subset \left[k\right]$ of *t* servers and any file index ι∈[n]. As each element of the vector g(ξ) is encoded separately from other elements, and corresponding random symbols are independent, each component of g(ξ) depends only on corresponding components of indicator vectors and is conditionally independent of everything else. Hence, our scheme is equivalent to the transmission over sn independent channels [23] and, as a result, we have

(4) $$\begin{array}{c} I\left(g\left(\beta_{l_{1}}\right),\ldots ,g\left(\beta_{l_{t}}\right);e_{1}^{\left(\iota \right)},\ldots ,e_{s}^{\left(\iota \right)}\right)\leq \sum_{i=1}^{sn}I\left(g{\left(\beta_{l_{1}}\right)}_{i},\ldots ,g{\left(\beta_{l_{t}}\right)}_{i};{\left(e_{1}^{\left(\iota \right)}\right)}_{i},\ldots ,{\left(e_{s}^{\left(\iota \right)}\right)}_{i}\right). \end{array}$$

It can be easily seen that for each *i*, $g{\left(\beta_{l_{1}}\right)}_{i},\ldots ,g{\left(\beta_{l_{t}}\right)}_{i}$ are *t* evaluations of a random polynomial $\psi_{i}$ of degree t+s−1 over $F_{q^{e}}$ at $l_{t}$ different points $\beta_{l_{1}},\ldots ,\beta_{l_{t}}$. Hence, for any given values of ${\left(e_{1}^{\left(\iota \right)}\right)}_{i},\ldots ,{\left(e_{s}^{\left(\iota \right)}\right)}_{i}$, using the Lagrange interpolation formula we can obtain a unique polynomial $\psi_{i}$ over $F_{q^{e}}$ such that $\psi_{i}\left(\alpha_{1}\right)={\left(e_{1}^{\left(\iota \right)}\right)}_{i},\ldots ,\psi_{i}\left(\alpha_{s}\right)={\left(e_{s}^{\left(\iota \right)}\right)}_{i}$ and $\psi_{i}\left(\beta_{l_{1}}\right)=g{\left(\beta_{l_{1}}\right)}_{i},\ldots ,\psi_{i}\left(\beta_{l_{t}}\right)=g{\left(\beta_{l_{t}}\right)}_{i}$. As a result, $I(g{\left(\beta_{l_{1}}\right)}_{i},\ldots ,g{\left(\beta_{l_{t}}\right)}_{i};{\left(e_{1}^{\left(\iota \right)}\right)}_{i},\ldots ,{\left(e_{s}^{\left(\iota \right)}\right)}_{i})=0$, and according to (4), the privacy property holds.

The property that responses from $k_{1}=r$ servers are enough for file retrieval trivially follows from the Lagrange interpolation formula and the fact that responses from servers are values of the polynomial ϕ over $F_{q^{e}}$ of degree r−1, so that $\phi \left(\alpha_{i}\right)=x_{i}^{\left(\iota \right)}$ for i∈[s]. Let us prove the correctness of scheme $\Pi_{1}$ for any $k_{d}$, d≠1 servers $\{l_{1},\ldots ,l_{k_{d}}\}=I$. It is clear that values $\left(\phi \left(\alpha_{1}\right),\ldots ,\phi \left(\alpha_{s}\right),\phi \left(\beta_{l_{1}}\right),\ldots ,\phi \left(\beta_{l_{k_{d}}}\right)\right)$ can be seen as a codeword of Reed–Solomon code of length $s+k_{d}$ and dimension *r*:

$$\begin{array}{c} {\mathrm{RS}}_{r}\left(\Omega_{\alpha }\cup \Omega_{\beta_{I}}\right)=\left\{\left(\phi \left(\alpha_{1}\right),\ldots ,\phi \left(\alpha_{s}\right),\phi \left(\beta_{l_{1}}\right),\ldots ,\phi \left(\beta_{l_{k_{d}}}\right)\right)|\phi \in F_{q^{e}}\left[\xi \right],\deg\left(\phi \right)<r\right\}, \end{array}$$

where $\Omega_{\beta_{I}}=\left\{\beta_{l_{1}},\ldots ,\beta_{l_{k_{d}}}\right\}$.

The dual of ${\mathrm{RS}}_{r}\left(\Omega_{\alpha }\cup \Omega_{\beta_{I}}\right)$ is a generalized Reed–Solomon code ${\mathrm{GRS}}_{k_{d}+s-r}\left(\Omega_{\alpha }\cup \Omega_{\beta_{I}}\right)$ ([25]), defined as follows:

(5) $$\begin{array}{cc} \; & {\mathrm{GRS}}_{k_{d}-t}\left(\Omega_{\alpha }\cup \Omega_{\beta_{I}}\right)=\\ \; & \left\{\left(u_{1}h^{\left(k_{d}\right)}\left(\alpha_{1}\right),\ldots ,u_{s}h^{\left(k_{d}\right)}\left(\alpha_{s}\right),v_{l_{1}}h^{\left(k_{d}\right)}\left(\beta_{l_{1}}\right),\ldots ,v_{l_{k_{d}}}h^{\left(k_{d}\right)}\left(\beta_{l_{k_{d}}}\right)\right)\right|\\ \; & h^{\left(k_{d}\right)}\in F_{q^{e}}\left[\xi \right],\deg\left(h^{\left(k_{d}\right)}\right)<k_{d}+s-r=k_{d}-t\}, \end{array}$$

where $u_{i}=\prod_{j\in [s]\setminus \{i\}}{\left(\alpha_{i}-\alpha_{j}\right)}^{-1}\times \prod_{o=1}^{k_{d}}{\left(\alpha_{i}-\beta_{l_{o}}\right)}^{-1}$ for i∈[s] and $v_{l_{j}}=\prod_{i=1}^{s}{\left(\beta_{l_{j}}-\alpha_{i}\right)}^{-1}\times \prod_{i\in I\setminus \{l_{j}\}}{\left(\beta_{l_{j}}-\beta_{i}\right)}^{-1}$ for $l_{j}\in I$.

Recall that $\alpha_{1},\ldots ,\alpha_{s}\in F_{q^{e}}$ are roots of distinct monic irreducible polynomials of degree *e* over $F_{q}$. Since $F_{q^{\lambda_{d}}}$ is a subfield of $F_{q^{e}}$, each element of $F_{q^{e}}$ can be represented as value of polynomial of degree less than $d=e/\lambda_{d}$ over $F_{q^{\lambda_{d}}}$ in point $\alpha_{i}$ for all i∈[s]. For each minimal polynomial ${\tilde{f}}_{j}^{\left(k_{d}\right)}\left(\xi \right)$ of $\alpha_{j}$ of degree *d* over $F_{q^{\lambda_{d}}}$ (see [26] for more details), we have

$$\left\{\begin{array}{cc} {\tilde{f}}_{j}^{\left(k_{d}\right)}\left(\alpha_{j}\right)=0\;\;\;{\tilde{f}}_{j}^{\left(k_{d}\right)}\left(\alpha_{i}\right)\neq 0\;\;\; & \mathrm{for}\;\;i\in [s],i\neq j\\ \prod_{j\in [s]\setminus \{i\}}{\tilde{f}}_{j}^{\left(k_{d}\right)}\left(\alpha_{m}\right)=0\;\;\; & \mathrm{for}\;\;m\in [s],m\neq i. \end{array}\right.$$

Let $\left\{\theta_{1}^{\left(k_{d}\right)},\ldots ,\theta_{d}^{\left(k_{d}\right)}\right\}$ be the basis of $F_{q^{e}}$ over $F_{q^{\lambda_{d}}}$, and $\left\{\eta_{1}^{\left(k_{d}\right)},\ldots ,\eta_{d}^{\left(k_{d}\right)}\right\}$ is its trace-orthogonal basis. For each δ∈[d] and i∈[s], we can represent the element $\frac{\eta_{\delta }^{\left(k_{d}\right)}}{u_{i}\prod_{j\in [s],j\setminus \{i\}}{\tilde{f}}_{j}^{\left(k_{d}\right)}\left(\alpha_{i}\right)}$ as the value of function $h_{i\delta }^{\left(k_{d}\right)}\left(\xi \right)\in F_{q^{\lambda_{d}}}\left[\xi \right]$ of degree less than *d* at point $\alpha_{i}$. It is clear that $\deg\left(h_{i\delta }^{\left(k_{d}\right)}\prod_{j\in [s]\setminus \{i\}}{\tilde{f}}_{j}\right)<sd\leq k_{d}-t$; hence, such functions belong to the dual generalized Reed–Solomon code (5). Also, we have

(6) $$h_{i\delta }^{\left(k_{d}\right)}\left(\alpha_{i}\right)\times \prod_{j\in [s]\setminus \{i\}}{\tilde{f}}_{j}^{\left(k_{d}\right)}\left(\alpha_{i}\right)=u_{i}^{-1}\eta_{\delta }^{\left(k_{d}\right)}$$

and

(7) $$h_{i\delta }^{\left(k_{d}\right)}\left(\alpha_{m}\right)\times \prod_{j\in [s]\setminus \{i\}}{\tilde{f}}_{j}^{\left(k_{d}\right)}\left(\alpha_{m}\right)=0\;\;\;\mathrm{for}\;\mathrm{all}\;\;m\in \left[s\right],m\neq i.$$

Consequently,

$$\begin{array}{cc} \; & (u_{1}h_{i\delta }^{\left(k_{d}\right)}\left(\alpha_{1}\right)\times \prod_{j\in [s]\setminus \{i\}}{\tilde{f}}_{j}^{\left(k_{d}\right)}\left(\alpha_{1}\right),\ldots ,u_{s}h_{i\delta }^{\left(k_{d}\right)}\left(\alpha_{s}\right)\times \prod_{j\in [s]\setminus \{i\}}{\tilde{f}}_{j}^{\left(k_{d}\right)}\left(\alpha_{s}\right),\\ \; & v_{l_{1}}h_{i\delta }^{\left(k_{d}\right)}\left(\beta_{l_{1}}\right)\times \prod_{j\in [s]\setminus \{i\}}{\tilde{f}}_{j}^{\left(k_{d}\right)}\left(\beta_{l_{1}}\right),\ldots ,v_{l_{k_{d}}}h_{i\delta }^{\left(k_{d}\right)}\left(\beta_{l_{k_{d}}}\right)\times \prod_{j\in [s]\setminus \{i\}}{\tilde{f}}_{j}^{\left(k_{d}\right)}\left(\beta_{l_{k_{d}}}\right))\cdot \\ \; & {\left(\phi \left(\alpha_{1}\right),\ldots ,\phi \left(\alpha_{s}\right),\phi \left(\beta_{l_{1}}\right),\ldots ,\phi \left(\beta_{l_{k_{d}}}\right)\right)}^{T}=0. \end{array}$$

Utilizing (6) and (7), we have

$$\begin{array}{cc} \; & \eta_{\delta }^{\left(k_{d}\right)}\phi \left(\alpha_{i}\right)+v_{l_{1}}h_{i\delta }^{\left(k_{d}\right)}\left(\beta_{l_{1}}\right)\phi \left(\beta_{l_{1}}\right)\times \prod_{j\in [s]\setminus \{i\}}{\tilde{f}}_{j}^{\left(k_{d}\right)}\left(\beta_{l_{1}}\right)+\ldots +\\ \; & v_{l_{k_{d}}}h_{i\delta }^{\left(k_{d}\right)}\left(\beta_{l_{k_{d}}}\right)\phi \left(\beta_{l_{k_{d}}}\right)\times \prod_{j\in [s]\setminus \{i\}}{\tilde{f}}_{j}^{\left(k_{d}\right)}\left(\beta_{l_{k_{d}}}\right)=0 \end{array}$$

and

(8) $$\begin{array}{c} \eta_{\delta }^{\left(k_{d}\right)}\phi \left(\alpha_{i}\right)=-\sum_{o=1}^{k_{d}}\left(v_{l_{o}}h_{i\delta }^{\left(k_{d}\right)}\left(\beta_{l_{o}}\right)\phi \left(\beta_{l_{o}}\right)\times \prod_{j\in [s]\setminus \{i\}}{\tilde{f}}_{j}^{\left(k_{d}\right)}\left(\beta_{l_{o}}\right)\right). \end{array}$$

Applying the trace-mapping function from $F_{q^{e}}$ to $F_{q^{\lambda_{d}}}$ to both sides of Equation (8), and utilizing the facts that $h_{i\delta }^{\left(k_{d}\right)}\left(\xi \right)\in F_{q^{\lambda_{d}}}\left[\xi \right]$, ${\tilde{f}}_{j}^{\left(k_{d}\right)}\left(\xi \right)\in F_{q^{\lambda_{d}}}\left[\xi \right]$, and $\beta_{l_{o}}\in F_{q}$ for all i,j∈[s], δ∈[d], $o\in [k_{d}]$, together with the linearity of the trace-mapping function, we obtain

$$\begin{array}{cc} \; & {\mathrm{Tr}}_{F_{q^{e}}/F_{q^{\lambda_{d}}}}\left(\eta_{\delta }^{\left(k_{d}\right)}\phi \left(\alpha_{i}\right)\right)=\\ \; & -\sum_{o=1}^{k_{d}}{\mathrm{Tr}}_{F_{q^{e}}/F_{q^{\lambda_{d}}}}\left(v_{l_{o}}\phi \left(\beta_{l_{o}}\right)h_{i\delta }^{\left(k_{d}\right)}\left(\beta_{l_{o}}\right)\times \prod_{j\in [s]\setminus \{i\}}{\tilde{f}}_{j}^{\left(k_{d}\right)}\left(\beta_{l_{o}}\right)\right)=\\ \; & -\sum_{o=1}^{k_{d}}h_{i\delta }^{\left(k_{d}\right)}\left(\beta_{l_{o}}\right)\left({\mathrm{Tr}}_{F_{q^{e}}/F_{q^{\lambda_{d}}}}\left(v_{l_{o}}\phi \left(\beta_{l_{o}}\right)\right)\times \prod_{j\in [s]\setminus \{i\}}{\tilde{f}}_{j}^{\left(k_{d}\right)}\left(\beta_{l_{o}}\right)\right). \end{array}$$

From the fact that $\left\{\theta_{1}^{\left(k_{d}\right)},\ldots ,\theta_{d}^{\left(k_{d}\right)}\right\}$ and $\left\{\eta_{1}^{\left(k_{d}\right)},\ldots ,\eta_{d}^{\left(k_{d}\right)}\right\}$ are trace-orthogonal bases of $F_{q^{e}}$ over $F_{q^{\lambda_{d}}}$, it is clear (see, for example, [22] [Ch. 2]) that

$$x_{i}^{\left(\iota \right)}=\phi \left(\alpha_{i}\right)=\sum_{\delta =1}^{d}\theta_{\delta }^{\left(k_{d}\right)}{\mathrm{Tr}}_{F_{q^{e}}/F_{q^{\lambda_{d}}}}\left(\eta_{\delta }^{\left(k_{d}\right)}\phi \left(\alpha_{i}\right)\right)$$

and, hence, all $\phi \left(\alpha_{1}\right),\ldots ,\phi \left(\alpha_{s}\right)$ can be retrieved by accessing ${\mathrm{Tr}}_{F_{q^{e}}/F_{q^{\lambda_{d}}}}\left(v_{l}\phi \left(\beta_{l}\right)\right)=a_{l}$ from all involved servers $l\in \left\{l_{1},\ldots ,l_{k_{d}}\right\}\subseteq \left[k\right]$. The observations that each file consists of *s* elements of $F_{q^{e}}$ for $e=\frac{\mathrm{lcm}(k_{2}-t,\ldots ,k_{\left|\Delta \right|}-t)}{s}$ and the download rate is equal to $\frac{k_{d}-t}{k_{d}}$ finish the proof. □

The proposed PIR scheme has a restricted number of servers $k_{d}$ participating in the retrieval process, namely, $\left(r-t\right)|\left(k_{d}-t\right)$. By letting s=r−t=1 in Scheme $\Pi_{1}$, we eliminate the restriction above and obtain the following corollary:

**Corollary** **1.** *In case of s=r−t=1, Scheme $\Pi_{1}$ is a k-server t-private PIR over $F_{q^{e}}$ with e=lcm(2,…,k−t), file size lcm(2,…,k−t)log(q) bits, and a retrieval threshold r that allows the retrieval from any r,r+1,…,k servers and achieves the asymptotic capacity (3) for b=0 for any r, so that t<r≤k≤q.*

## 3. Byzantine-Resistant PIR Scheme

Let us construct a *k*-server PIR scheme with *t*-colluding and *b*-Byzantine servers by modifying the construction from Section 2.3. To deal with Byzantine servers, inspired by [21], we modify the protocol by multiplying the responses by specifically selected polynomials so that the server responses form a generalized Reed–Solomon code. Importantly, to ensure the possibility of error correction, we increase the number of required responses so that the codeword formed by them possesses the desired code-distance properties. For the convenience of the reader, before giving the details of the whole scheme and providing a proof of its correctness, let us illustrate the repair process using the trace-repair framework from $k_{d}=ds+t+2b$ servers in the presence of up to *b* incorrect answers from servers.

The pre-computation and download steps remain the same as for the scheme with correct answers from servers. To find the file of interest, before trace reconstruction, we form the following vector from the received traces $\left(\prod_{j=1}^{s}{\tilde{f}}_{j}^{\left(k_{d}\right)}\left(\beta_{l_{1}}\right)a_{1},\ldots ,\prod_{j=1}^{s}{\tilde{f}}_{j}^{\left(k_{d}\right)}\left(\beta_{l_{k_{d}}}\right)a_{k_{d}}\right)$ and decode it as a codeword of a generalized Reed–Solomon code using any decoding algorithm (see, for example, [25]). As a result, we obtain the correct values of the elements of the vector above and can recover the correct values $a_{1},\ldots ,a_{k_{d}}$. Further steps remain the same.

*Scheme $\Pi_{2}$: k*-server* t*-private *b*-Byzantine-resistant PIR    Let *t*, *r*, $k_{d}$, and *k* be positive integers satisfying $t<r-2b\leq k_{d}-2b\leq k\leq q$, s=r−t−2b, $k_{d}\in \Delta =\left\{k_{1},\ldots ,k_{\left|\Delta \right|}\right\},$ where $k_{d}=ds+t+2b\leq k$ and $d\in N^{*}$. Denote by $e≜\frac{\mathrm{lcm}(k_{2}-t-2b,\ldots ,k_{\left|\Delta \right|}-t-2b)}{s}$. Let $\Omega_{\alpha }=\left\{\alpha_{1},\ldots ,\alpha_{s}\right\}\subset F_{q^{e}}$, $\Omega_{\chi }=\left\{\chi_{1},\ldots ,\chi_{t}\right\}\subset F_{q^{e}}$, and $\Omega_{\beta }=\left\{\beta_{1},\ldots ,\beta_{k}\right\}\subseteq F_{q}$ be publicly known non-intersecting sets such that all elements of $\Omega_{\alpha }$ are roots of distinct monic irreducible polynomials of degree *e* over $F_{q}$.
*Query Generation Algorithm:* To retrieve the file ι∈[n], the user generates *t* random vectors $r^{\left(1\right)},\ldots ,r^{\left(t\right)}\in F_{q^{e}}^{sn}$ and draws a random degree-(t+s−1) curve$$\begin{array}{cc} g(\xi )\; & ≜\sum_{o=1}^{s}\prod_{p\in [s]\setminus \{o\}}\left(\frac{\xi -\alpha_{p}}{\alpha_{o}-\alpha_{p}}\right)\prod_{p=1}^{t}\left(\frac{\xi -\chi_{p}}{\alpha_{o}-\chi_{p}}\right)e_{o}^{\left(\iota \right)}\\ \; & +\sum_{o=1}^{t}\prod_{p=1}^{s}\left(\frac{\xi -\alpha_{p}}{\chi_{o}-\alpha_{p}}\right)\prod_{p\in [t]\setminus \{o\}}\left(\frac{\xi -\chi_{p}}{\chi_{o}-\chi_{p}}\right)r^{\left(o\right)} \end{array}$$
that resides in $F_{q^{e}}^{sn}$ and passes through points $\left(\alpha_{1},e_{1}^{\left(\iota \right)}\right),\ldots ,\left(\alpha_{s},e_{s}^{\left(\iota \right)}\right)$. The query to server l∈[k] is $g(\beta_{l})$. We note that g depends on the retrieved index ι, but we omit the subscript ι for readability. As introduced earlier, $e_{i}^{\left(\iota \right)}$ is an indicator vector of length sn with all zeros except for a single one at position s(ι−1)+i.*Answer Generation Algorithm:* Upon receiving the query $g(\beta_{l})$, server l∈[k] computes $F_{x}\left(g\left(\beta_{l}\right)\right)$, where $F_{x}$ is the database polynomial over $F_{q^{e}}$ determined in (1). We can observe that $F_{x}\left(g\left(\xi \right)\right)=\phi \left(\xi \right)$, which is a polynomial in ξ of degree s+t−1=r−2b−1 such that $\phi \left(\alpha_{i}\right)=F_{x}\left(g\left(\alpha_{i}\right)\right)=F_{x}\left(e_{i}^{\left(\iota \right)}\right)=x_{i}^{\left(\iota \right)}$ for i∈[s].
-Each involved server l∈I, $\left|I\right|=k_{d}$ responds with$$a_{l}={\mathrm{Tr}}_{F_{q^{e}}/F_{q^{\lambda_{d}}}}\left(v_{l}\phi \left(\beta_{l}\right)\right)\in F_{q^{\lambda_{d}}},$$
where $\lambda_{d}=\frac{es}{k_{d}-t-2b}$ and$$v_{l}=\left\{\begin{array}{cc} 1 & \mathrm{if}\;d=1\\ \prod_{j=1}^{s}{\left(\beta_{l}-\alpha_{j}\right)}^{-1}\times \prod_{j\in I\setminus \{l\}}{\left(\beta_{l}-\beta_{j}\right)}^{-1} & \mathrm{otherwise}. \end{array}\right.$$Clearly, when d=1, we have ${\mathrm{Tr}}_{F_{q^{e}}/F_{q^{\lambda_{d}}}}\left(x\right)=x$ and, as a result, $a_{l}=\phi \left(\beta_{l}\right)$.*File Retrieval Algorithm*-For retrieval using answers from $k_{1}=r$ servers, the user applies a Reed–Solomon decoding algorithm to recover the polynomial ϕ(ξ) and, as a result, obtains $\phi \left(\alpha_{1}\right),\ldots ,\phi \left(\alpha_{s}\right)$ (see, for example, [25]).-For retrieval using answers from the set I⊆[k], $\left|I\right|=k_{d}$, d≠1, the user decodes the vector$$\begin{array}{c} \left(\prod_{j=1}^{s}{\tilde{f}}_{j}^{\left(k_{d}\right)}\left(\beta_{l_{1}}\right){\mathrm{Tr}}_{F_{q^{e}}/F_{q^{\lambda_{d}}}}\left(v_{l_{1}}\phi \left(\beta_{l_{1}}\right)\right),\ldots ,\prod_{j=1}^{s}{\tilde{f}}_{j}^{\left(k_{d}\right)}\left(\beta_{l_{k_{d}}}\right){\mathrm{Tr}}_{F_{q^{e}}/F_{q^{\lambda_{d}}}}\left(v_{l_{k_{d}}}\phi \left(\beta_{l_{k_{d}}}\right)\right)\right), \end{array}$$
where ${\tilde{f}}_{j}^{\left(k_{d}\right)}\left(\xi \right)$ is the minimal polynomial of $\alpha_{j}$ over $F_{q^{\lambda_{d}}}$, as a codeword of generalized Reed–Solomon code over $F_{q^{\lambda_{d}}}$, using any decoding algorithm (see, for example, [25]), and extracts the correct values of ${\mathrm{Tr}}_{F_{q^{e}}/F_{q^{\lambda_{d}}}}\left(v_{l}\phi \left(\beta_{l}\right)\right)$ for l∈I.Next, the user prepares a basis $\left\{\theta_{1}^{\left(k_{d}\right)},\ldots ,\theta_{d}^{\left(k_{d}\right)}\right\}$ for $F_{q^{e}}$ over $F_{q^{\lambda_{d}}}$ and its trace-orthogonal basis $\left\{\eta_{1}^{\left(k_{d}\right)},\ldots ,\eta_{d}^{\left(k_{d}\right)}\right\}$. After that, for all i∈[s] and δ∈[d], the user chooses polynomials $h_{i\delta }^{\left(k_{d}\right)}\left(\xi \right)\in F_{q^{\lambda_{d}}}\left[\xi \right]$ of degree less than *d* so that$$h_{i\delta }^{\left(k_{d}\right)}\left(\alpha_{i}\right)=\frac{\eta_{\delta }^{\left(k_{d}\right)}}{u_{i}\prod_{j\in [s]\setminus \{i\}}{\tilde{f}}_{j}^{\left(k_{d}\right)}\left(\alpha_{i}\right)},$$
where ${\tilde{f}}_{j}^{\left(k_{d}\right)}\left(\xi \right)$ is the minimal polynomial of $\alpha_{j}$ over $F_{q^{\lambda_{d}}}$ and$$u_{i}=\prod_{j\in [s]\setminus \{i\}}{\left(\alpha_{i}-\alpha_{j}\right)}^{-1}\times \prod_{l\in I}{\left(\alpha_{i}-\beta_{l}\right)}^{-1}.$$The user retrieves the file of interest by$$\begin{array}{c} x_{i}^{\left(\iota \right)}=\phi \left(\alpha_{i}\right)=-\sum_{\delta =1}^{d}\theta_{\delta }^{\left(k_{d}\right)}\left(\sum_{l\in I}h_{i\delta }^{\left(k_{d}\right)}\left(\beta_{l}\right)a_{l}\times \prod_{j\in [s]\setminus \{i\}}{\tilde{f}}_{j}^{\left(k_{d}\right)}\left(\beta_{l}\right)\right), \end{array}$$
for all i∈[s].

**Theorem** **2.** Scheme $\Pi_{2}$ is a k-server t-private b-Byzantine-resistant PIR over $F_{q^{e}}$ with retrieval threshold $k_{1}=r$ and file size $\mathrm{lcm}\left(k_{2}-t-2b,\ldots ,k_{\left|\Delta \right|}-t-2b\right)\log\left(q\right)$ that allows the retrieval from any $k_{1},\ldots ,k_{\left|\Delta \right|}$ servers and achieves the asymptotic capacity (3) for any given s and r so that $t<r\leq k_{d}\leq k\leq q$, s=r−t−2b and $k_{d}\in \Delta =\left\{k_{1},\ldots ,k_{\left|\Delta \right|}\right\}$ for $k_{d}=ds+t+2b$, $d\in N^{*}$ and $e=\frac{\mathrm{lcm}(k_{2}-t-2b,\ldots ,k_{\left|\Delta \right|}-t-2b)}{s}$.

**Proof.**  Let us prove the privacy and correctness properties according to Definition 1. The proof of privacy is identical to that of Theorem 1 and is therefore omitted here. We will now demonstrate the correctness property, beginning with the case where $k_{1}=r$. The property that responses from $k_{1}=r$ servers are enough for file retrieval follows from the fact that values $\left(\phi \left(\alpha_{1}\right),\ldots ,\phi \left(\alpha_{s}\right),\phi \left(\beta_{l_{1}}\right),\ldots ,\phi \left(\beta_{l_{k_{1}}}\right)\right)$ can be seen as a codeword of Reed–Solomon code of length $k_{1}+s$ and dimension r−2b:

$$\begin{array}{c} {\mathrm{RS}}_{r-2b}\left(\Omega_{\alpha }\cup \Omega_{\beta }\right)=\left\{\left(\phi \left(\alpha_{1}\right),\ldots ,\phi \left(\alpha_{s}\right),\phi \left(\beta_{l_{1}}\right),\ldots ,\phi \left(\beta_{l_{k_{1}}}\right)\right)|\phi \in F_{q^{e}}\left[\xi \right],\deg\left(\phi \right)<r-2b\right\}. \end{array}$$

As a result, with values of the polynomial ϕ in any $k_{1}=r$ points, we can correctly interpolate it in the presence of *b* incorrect values utilizing a Reed–Solomon decoding algorithm (see, for example, [25]).

Let us prove the correctness of scheme $\Pi_{2}$ for the case of $k_{d}$, d≠1 servers $\{l_{1},\ldots ,l_{k_{d}}\}=I$. It is clear that values $\left(\phi \left(\alpha_{1}\right),\ldots ,\phi \left(\alpha_{s}\right),\phi \left(\beta_{l_{1}}\right),\ldots ,\phi \left(\beta_{l_{k_{d}}}\right)\right)$ can be seen as a codeword of Reed–Solomon code of length $k_{d}+s$ and dimension r−2b:

$$\begin{array}{c} {\mathrm{RS}}_{r-2b}\left(\Omega_{\alpha }\cup \Omega_{\beta_{I}}\right)=\left\{\left(\phi \left(\alpha_{1}\right),\ldots ,\phi \left(\alpha_{s}\right),\phi \left(\beta_{l_{1}}\right),\ldots ,\phi \left(\beta_{l_{k_{d}}}\right)\right)|\phi \in F_{q^{e}}\left[\xi \right],\deg\left(\phi \right)<r-2b\right\}, \end{array}$$

where $\Omega_{\beta_{I}}=\left\{\beta_{l_{1}},\ldots ,\beta_{l_{k_{d}}}\right\}$.

The dual of ${\mathrm{RS}}_{r-2b}\left(\Omega_{\alpha }\cup \Omega_{\beta_{I}}\right)$ is a generalized Reed–Solomon code ${\mathrm{GRS}}_{k_{d}-t}\left(\Omega_{\alpha }\cup \Omega_{\beta_{I}}\right)$, defined as follows:

(9) $$\begin{array}{cc} \; & {\mathrm{GRS}}_{k_{d}-t}\left(\Omega_{\alpha }\cup \Omega_{\beta_{I}}\right)=\{(u_{1}h^{\left(k_{d}\right)}\left(\alpha_{1}\right),\ldots ,u_{s}h^{\left(k_{d}\right)}\left(\alpha_{s}\right),\\ \; & v_{l_{1}}h^{\left(k_{d}\right)}\left(\beta_{l_{1}}\right),\ldots ,v_{l_{k_{d}}}h^{\left(k_{d}\right)}\left(\beta_{l_{k_{d}}}\right))|h^{\left(k_{d}\right)}\in F_{q^{e}}\left[\xi \right],\\ \; & \deg\left(h^{\left(k_{d}\right)}\right)<k_{d}+s-r+2b=k_{d}-t\}, \end{array}$$

where $u_{i}=\prod_{j\in [s]\setminus \{i\}}{\left(\alpha_{i}-\alpha_{j}\right)}^{-1}\times \prod_{o=1}^{k_{d}}{\left(\alpha_{i}-\beta_{l_{o}}\right)}^{-1}$ for i∈[s] and $v_{l_{j}}=\prod_{i=1}^{s}{\left(\beta_{l_{j}}-\alpha_{i}\right)}^{-1}\times \prod_{i\in I\setminus \{l_{j}\}}{\left(\beta_{l_{j}}-\beta_{i}\right)}^{-1}$ for $l_{j}\in I$.

Recall that $\alpha_{1},\ldots ,\alpha_{s}\in F_{q^{e}}$ are roots of distinct monic irreducible polynomials of degree *e* over $F_{q}$. Since $F_{q^{\lambda_{d}}}$ is a subfield of $F_{q^{e}}$, each element of $F_{q^{e}}$ can be represented as a polynomial value of degree less than $d=e/\lambda_{d}$ over $F_{q^{\lambda_{d}}}$ in point $\alpha_{i}$ for all i∈[s]. For each minimal polynomial ${\tilde{f}}_{j}^{\left(k_{d}\right)}$ of $\alpha_{j}$ of degree *d* over $F_{q^{\lambda_{d}}}$ (see [26] for more details), we have

$$\left\{\begin{array}{cc} {\tilde{f}}_{j}^{\left(k_{d}\right)}\left(\alpha_{j}\right)=0\;\;\;{\tilde{f}}_{j}^{\left(k_{d}\right)}\left(\alpha_{i}\right)\neq 0\;\;\; & \mathrm{for}\;\;i\in [s],i\neq j\\ \prod_{j\in [s]\setminus \{i\}}{\tilde{f}}_{j}^{\left(k_{d}\right)}\left(\alpha_{m}\right)=0\;\;\; & \mathrm{for}\;\;m\in [s],m\neq i. \end{array}\right.$$

Let $\left\{\theta_{1}^{\left(k_{d}\right)},\ldots ,\theta_{d}^{\left(k_{d}\right)}\right\}$ be the basis of $F_{q^{e}}$ over $F_{q^{\lambda_{d}}}$, and let $\left\{\eta_{1}^{\left(k_{d}\right)},\ldots ,\eta_{d}^{\left(k_{d}\right)}\right\}$ be its trace-orthogonal basis. For each δ∈[d] and i∈[s], we can represent the element $\frac{\eta_{\delta }^{\left(k_{d}\right)}}{u_{i}\prod_{j\in [s]\setminus \{i\}}{\tilde{f}}_{j}^{\left(k_{d}\right)}\left(\alpha_{i}\right)}$ as the value of function $h_{i\delta }^{\left(k_{d}\right)}\left(\xi \right)\in F_{q^{\lambda_{d}}}\left[\xi \right]$ of degree less than *d* at point $\alpha_{i}$. It is clear that $\deg\left(h_{i\delta }^{\left(k_{d}\right)}\prod_{j\in [s]\setminus \{i\}}{\tilde{f}}_{j}\right)<sd<k_{d}-t$; hence, such functions belong to the dual generalized Reed–Solomon code (9). Also, we have

(10) $$h_{i\delta }^{\left(k_{d}\right)}\left(\alpha_{i}\right)\times \prod_{j\in [s]\setminus \{i\}}{\tilde{f}}_{j}^{\left(k_{d}\right)}\left(\alpha_{i}\right)=u_{i}^{-1}\eta_{\delta }^{\left(k_{d}\right)}$$

and

(11) $$h_{i\delta }^{\left(k_{d}\right)}\left(\alpha_{m}\right)\prod_{j\in [s]\setminus \{i\}}{\tilde{f}}_{j}^{\left(k_{d}\right)}\left(\alpha_{m}\right)=0\;\;\;\mathrm{for}\mathrm{all}\;\;m\in \left[s\right],m\neq i.$$

Consequently,

$$\begin{array}{cc} \; & (u_{1}h_{i\delta }^{\left(k_{d}\right)}\left(\alpha_{1}\right)\times \prod_{j\in [s]\setminus \{i\}}{\tilde{f}}_{j}^{\left(k_{d}\right)}\left(\alpha_{1}\right),\ldots ,u_{s}h_{i\delta }^{\left(k_{d}\right)}\left(\alpha_{s}\right)\times \prod_{j\in [s]\setminus \{i\}}{\tilde{f}}_{j}^{\left(k_{d}\right)}\left(\alpha_{s}\right),\\ \; & v_{l_{1}}h_{i\delta }^{\left(k_{d}\right)}\left(\beta_{l_{1}}\right)\times \prod_{j\in [s]\setminus \{i\}}{\tilde{f}}_{j}^{\left(k_{d}\right)}\left(\beta_{l_{1}}\right),\ldots ,v_{l_{k_{d}}}h_{i\delta }^{\left(k_{d}\right)}\left(\beta_{l_{k_{d}}}\right)\times \prod_{j\in [s]\setminus \{i\}}{\tilde{f}}_{j}^{\left(k_{d}\right)}\left(\beta_{l_{k_{d}}}\right))\cdot \\ \; & {\left(\phi \left(\alpha_{1}\right),\ldots ,\phi \left(\alpha_{s}\right),\phi \left(\beta_{l_{1}}\right),\ldots ,\phi \left(\beta_{l_{k_{d}}}\right)\right)}^{T}=0. \end{array}$$

Employing (10) and (11), we have

$$\begin{array}{cc} \; & \eta_{\delta }^{\left(k_{d}\right)}\phi \left(\alpha_{i}\right)+v_{l_{1}}h_{i\delta }^{\left(k_{d}\right)}\left(\beta_{l_{1}}\right)\phi \left(\beta_{l_{1}}\right)\times \prod_{j\in [s]\setminus \{i\}}{\tilde{f}}_{j}^{\left(k_{d}\right)}\left(\beta_{l_{1}}\right)+\ldots +\\ \; & v_{l_{k_{d}}}h_{i\delta }^{\left(k_{d}\right)}\left(\beta_{l_{k_{d}}}\right)\phi \left(\beta_{l_{k_{d}}}\right)\times \prod_{j\in [s]\setminus \{i\}}{\tilde{f}}_{j}^{\left(k_{d}\right)}\left(\beta_{l_{k_{d}}}\right)=0 \end{array}$$

and

(12) $$\begin{array}{c} \eta_{\delta }^{\left(k_{d}\right)}\phi \left(\alpha_{i}\right)=-\sum_{o=1}^{k_{d}}\left(v_{l_{o}}h_{i\delta }^{\left(k_{d}\right)}\left(\beta_{l_{o}}\right)\phi \left(\beta_{l_{o}}\right)\times \prod_{j\in [s]\setminus \{i\}}{\tilde{f}}_{j}^{\left(k_{d}\right)}\left(\beta_{l_{o}}\right)\right). \end{array}$$

Applying the trace-mapping function from $F_{q^{e}}$ to $F_{q^{\lambda_{d}}}$ to both sides of Equation (12), and utilizing the facts that $h_{i\delta }^{\left(k_{d}\right)}\left(\xi \right)\in F_{q^{\lambda_{d}}}\left[\xi \right]$, ${\tilde{f}}_{j}^{\left(k_{d}\right)}\left(\xi \right)\in F_{q^{\lambda_{d}}}\left[\xi \right]$, and $\beta_{l_{o}}\in F_{q}$ for all i,j∈[s], δ∈[d], $o\in [k_{d}]$, together with the linearity of the trace-mapping function, we obtain

$$\begin{array}{cc} \; & {\mathrm{Tr}}_{F_{q^{e}}/F_{q^{\lambda_{d}}}}\left(\eta_{\delta }^{\left(k_{d}\right)}\phi \left(\alpha_{i}\right)\right)=-\sum_{o=1}^{k_{d}}{\mathrm{Tr}}_{F_{q^{e}}/F_{q^{\lambda_{d}}}}\left(v_{l_{o}}\phi \left(\beta_{l_{o}}\right)h_{i\delta }^{\left(k_{d}\right)}\left(\beta_{l_{o}}\right)\times \prod_{j\in [s]\setminus \{i\}}{\tilde{f}}_{j}^{\left(k_{d}\right)}\left(\beta_{l_{o}}\right)\right)\\ \; & =-\sum_{o=1}^{k_{d}}h_{i\delta }^{\left(k_{d}\right)}\left(\beta_{l_{o}}\right)\cdot \left({\mathrm{Tr}}_{F_{q^{e}}/F_{q^{\lambda_{d}}}}\left(v_{l_{o}}\phi \left(\beta_{l_{o}}\right)\right)\times \prod_{j\in [s]\setminus \{i\}}{\tilde{f}}_{j}^{\left(k_{d}\right)}\left(\beta_{l_{o}}\right)\right). \end{array}$$

From the fact that $\left\{\theta_{1}^{\left(k_{d}\right)},\ldots ,\theta_{d}^{\left(k_{d}\right)}\right\}$ and $\left\{\eta_{1}^{\left(k_{d}\right)},\ldots ,\eta_{d}^{\left(k_{d}\right)}\right\}$ are trace-orthogonal bases of $F_{q^{e}}$ over $F_{q^{\lambda_{d}}}$, it is clear (see, for example, [22] [Ch. 2]) that

$$x_{i}^{\left(\iota \right)}=\phi \left(\alpha_{i}\right)=\sum_{\delta =1}^{d}\theta_{\delta }^{\left(k_{d}\right)}{\mathrm{Tr}}_{F_{q^{e}}/F_{q^{\lambda_{d}}}}\left(\eta_{\delta }^{\left(k_{d}\right)}\phi \left(\alpha_{i}\right)\right)$$

and, hence, all $\phi \left(\alpha_{1}\right),\ldots ,\phi \left(\alpha_{s}\right)$ can be recovered by accessing ${\mathrm{Tr}}_{F_{q^{e}}/F_{q^{\lambda_{d}}}}\left(v_{l}\phi \left(\beta_{l}\right)\right)=a_{l}$ from all involved servers $l\in \left\{l_{1},\ldots ,l_{k_{d}}\right\}\subseteq \left[k\right]$.

Let us show that we can correctly recover $\phi \left(\alpha_{1}\right),\ldots ,\phi \left(\alpha_{s}\right)$ in case of at most *b* incorrect values of ${\mathrm{Tr}}_{F_{q^{e}}/F_{q^{\lambda_{d}}}}\left(v_{l}\phi \left(\beta_{l}\right)\right)$. The main idea here is to formally show that the elements $\prod_{j\in [s]}{\tilde{f}}_{j}^{\left(k_{d}\right)}\left(\beta_{l_{o}}\right){\mathrm{Tr}}_{F_{q^{e}}/F_{q^{\lambda_{d}}}}\left(v_{l_{o}}\phi \left(\beta_{l_{o}}\right)\right)$ form a codeword of a generalized Reed–Solomon code over $F_{q^{\lambda_{d}}}$ of length $k_{d}$ and dimension $k_{d}-2b$. To do so, let us define the functions ${\tilde{h}}_{\epsilon }^{\left(k_{d}\right)}\left(\xi \right)=\xi^{\epsilon }\prod_{j\in [s]}{\tilde{f}}_{j}^{\left(k_{d}\right)}\left(\xi \right)\in F_{q^{\lambda_{d}}}\left[\xi \right]$.

It is clear that ${\tilde{h}}_{\epsilon }^{\left(k_{d}\right)}\left(\alpha_{i}\right)=0$ for all i∈[s] and $\deg(h_{\epsilon }^{\left(k_{d}\right)}\left(\xi \right))<sd+\epsilon$. Hence, for all ϵ<2b, we know that $\deg\left({\tilde{h}}_{\epsilon }^{\left(k_{d}\right)}\left(\xi \right)\right)<k_{d}-t$ and, as a result, these functions belong to the generalized Reed–Solomon code (9), which is dual to the original Reed–Solomon code. We can write down the duality condition

(13) $$\begin{array}{cc} \; & \left(u_{1}{\tilde{h}}_{\epsilon }^{\left(k_{d}\right)}\left(\alpha_{1}\right),\ldots ,u_{s}{\tilde{h}}_{\epsilon }^{\left(k_{d}\right)}\left(\alpha_{s}\right),v_{l_{1}}{\tilde{h}}_{\epsilon }^{\left(k_{d}\right)}\left(\beta_{l_{1}}\right),\ldots ,v_{l_{k_{d}}}{\tilde{h}}_{\epsilon }^{\left(k_{d}\right)}\left(\beta_{l_{k_{d}}}\right)\right)\\ \; & \cdot {\left(\phi \left(\alpha_{1}\right),\ldots ,\phi \left(\alpha_{s}\right),\phi \left(\beta_{l_{1}}\right),\ldots ,\phi \left(\beta_{l_{k_{d}}}\right)\right)}^{T}=v_{l_{1}}{\tilde{h}}_{\epsilon }^{\left(k_{d}\right)}\left(\beta_{l_{1}}\right)\phi \left(\beta_{l_{1}}\right)+\ldots +v_{l_{k_{d}}}{\tilde{h}}_{\epsilon }^{\left(k_{d}\right)}\left(\beta_{l_{k_{d}}}\right)\phi \left(\beta_{l_{k_{d}}}\right)=0. \end{array}$$

As ${\tilde{h}}_{\epsilon }^{\left(k_{d}\right)}\left(\xi \right)\in F_{q^{\lambda_{d}}}\left[\xi \right]$ and $\left\{\beta_{l_{1}},\ldots ,\beta_{l_{k_{d}}}\right\}\subseteq F_{q}$, applying the trace-mapping function from $F_{q^{e}}$ to $F_{q^{\lambda_{d}}}$ to both sides of Equation (13) and utilizing its linearity, we have

$$\begin{array}{cc} \; & \beta_{l_{1}}^{\epsilon }\prod_{j\in [s]}{\tilde{f}}_{j}^{\left(k_{d}\right)}\left(\beta_{l_{1}}\right){\mathrm{Tr}}_{F_{q^{e}}/F_{q^{\lambda_{d}}}}\left(v_{l_{1}}\phi \left(\beta_{l_{1}}\right)\right)+\ldots +\beta_{l_{k_{d}}}^{\epsilon }\prod_{j\in [s]}{\tilde{f}}_{j}^{\left(k_{d}\right)}\left(\beta_{l_{k_{d}}}\right){\mathrm{Tr}}_{F_{q^{e}}/F_{q^{\lambda_{d}}}}\left(v_{l_{k_{d}}}\phi \left(\beta_{l_{k_{d}}}\right)\right)=0, \end{array}$$

where ϵ=0,1,…,2b−1, and all multipliers in each term belong to $F_{q^{\lambda_{d}}}$ for all $o\in [k_{d}]$. Applying [21] [Proposition 1], we find that the elements $\prod_{j\in [s]}{\tilde{f}}_{j}^{\left(k_{d}\right)}\left(\beta_{l_{o}}\right){\mathrm{Tr}}_{F_{q^{e}}/F_{q^{\lambda_{d}}}}\left(v_{l_{o}}\phi \left(\beta_{l_{o}}\right)\right)$ form a codeword of generalized Reed–Solomon code over $F_{q^{\lambda_{d}}}$ that can correct up to *b* errors, and users can retrieve the correct values of ${\mathrm{Tr}}_{F_{q^{e}}/F_{q^{\lambda_{d}}}}\left(v_{l_{o}}\phi \left(\beta_{l_{o}}\right)\right)$ from server responses for all $o\in [k_{d}]$ by employing any generalized Reed–Solomon decoder (see, for example [25]). The observations that each file consists of *s* elements of $F_{q^{e}}$ for $e=\frac{\mathrm{lcm}(k_{2}-t-2b,\ldots ,k_{\left|\Delta \right|}-t-2b)}{s}$ and that the download rate is equal to $\frac{k_{d}-t-2b}{k_{d}}$ finish the proof. □

The proposed PIR scheme has a restricted number of servers participating in the retrieval process, namely, $\left(r-t-2b\right)|\left(k_{d}-t-2b\right)$. By letting s=r−t−2b=1 in Scheme $\Pi_{2}$, we eliminate the restriction above and obtain the following corollary:

**Corollary** **2.** *In case of s=r−t−2b=1, Scheme $\Pi_{2}$ is a k-server t-private b-Byzantine-resistant PIR over $F_{q^{e}}$ with e=lcm(2,…,k−t−2b), file size lcm(2,…,k−t−2b)log(q) bits, and a retrieval threshold r that allows the retrieval from any r,r+1,…,k servers and achieves the asymptotic capacity (3) for any r so that t<r−2b≤k−2b<k≤q.*

## 4. Lower Bound on the File Size

In this section, following the derivations in [20] [Section V], we describe the class of Byzantine-resistant PIR schemes and demonstrate that our schemes belong to this class and achieve optimal file size. In what follows, we consider a *k*-server *b*-Byzantine-resistant PIR scheme with a retrieval threshold *r*, deployed over the extended field $F_{q^{e}}$ with base field size q≥k and sub-packetization *s*. We omit these details within this section for clarity purposes.

**Definition** **2** (Balanced Byzantine-resistant PIR)**.** *The Byzantine-resistant PIR scheme is balanced if, for any permitted set of involved servers I⊆[k] with |I|≥r, the user downloads a single element of the same subfield $F_{q^{R_{I}}}$ from each involved server.*

**Definition** **3** (Rate-optimal Byzantine-resistant PIR)**.** *The Byzantine-resistant PIR scheme is rate-optimal if, for any file $f^{\left(\iota \right)}$, we have $H\left(f^{\left(\iota \right)}\right)=\left(r-2b-t\right)e=se$.*

After introducing the necessary definitions, we can formulate the main theorem of this section:

**Theorem** **3.** *For a balanced rate-optimal Byzantine-resistant PIR scheme that achieves the asymptotic capacity (3) and admits the retrieval from any $k_{d}\in \Delta =\left\{k_{1},\ldots ,k_{\left|\Delta \right|}\right\}$ servers with the retrieval threshold $k_{1}=r$, the following hold:*

$$\left(r-2b-t\right)|\left(k_{d}-2b-t\right)\;\;\;\mathrm{for}\;\;\mathrm{any}\;\;k_{d}\in \Delta$$

$$se\geq \mathrm{lcm}(k_{2}-2b-t,\ldots ,k_{\left|\Delta \right|}-2b-t).$$

**Proof.**  As the considered scheme is rate-optimal, s=r−2b−t. Since the considered scheme is balanced, we assume that the user downloads a single element of $F_{q^{R_{I}}}$ from each involved server l∈I, $\left|I\right|=k_{d}$. At the same time, the scheme achieves the asymptotic capacity (3), and the download cost is optimal. By writing this down along with the total amount of data downloaded from the servers, we can state the following equality:

$$k_{d}\log_{\begin{array}{c} q \end{array}}q^{R_{I}}=\frac{k_{d}}{k_{d}-2b-t}se.$$

This leads to $se=R_{I}\left(k_{d}-2b-t\right)$. Since *e* is a positive integer and $F_{q^{R_{I}}}$ is a subfield of $F_{q^{e}}$, then $R_{I}$ divides *e* and, hence,

(14) $$\left(r-2b-t\right)|\left(k_{d}-2b-t\right)\;\;\;\;\;\mathrm{and}\;\;\;\;\;\frac{k_{d}-2b-t}{r-2b-t}|e$$

Since Equation (14) holds for all $k_{d}\in \Delta =\left\{k_{1},\ldots ,k_{\left|\Delta \right|}\right\}$ and $k_{1}=r$, then $\frac{\mathrm{lcm}(k_{2}-2b-t,\ldots ,k_{\left|\Delta \right|}-2b-t)}{r-2b-t}|e$ and, as a result,

$$\begin{array}{c} e\geq \frac{\mathrm{lcm}(k_{2}-2b-t,\ldots ,k_{\left|\Delta \right|}-2b-t)}{r-2b-t}=\frac{\mathrm{lcm}(k_{2}-2b-t,\ldots ,k_{\left|\Delta \right|}-2b-t)}{s}. \end{array}$$

□

**Corollary** **3.** *The scheme $\Pi_{2}$ is a balanced capacity-achieving rate-optimal Byzantine-resistant PIR with optimal file size for an asymptotically large number of files.*

## 5. Comparison

In this section, we compare PIR schemes $\Pi_{1}$ and $\Pi_{2}$ in the case where s=1 with Staircase-PIR from [27]. Staircase-PIR allows retrieval without restrictions on the number of participating servers and achieves the asymptotic capacity given in (3). It also does not have a sub-packetization level exponential in the number of files *n*, as in the previous capacity-achieving scheme [7], making it the best scheme for comparison. We denote the latter as scheme $A_{1}$ and present its parameters in the following theorem:

**Theorem** **4.** *Scheme $A_{1}$ is k-server t-private PIR over $F_{q}$ with file size $\left(r-t\right)\mathrm{lcm}\left(k_{2}-t,\ldots ,k_{d}-t\right)\log\left(q\right)$ bits and retrieval threshold $k_{1}=r$ that allows retrieval from any r,r+1,…,k servers and achieves the asymptotic capacity (3) for b=0 and any given r so that $t<r<k_{2}<\ldots <k_{d}=k\leq q$.*

Scheme $A_{1}$ assumes that the servers are honest-but-curious and provide correct answers. To add the *b*-Byzantine resistance, we can utilize error-correction capabilities of underlining staircase codes in the same way as shown in [28]. We present the parameters of the resulting scheme $A_{2}$ in the following theorem:

**Theorem** **5.** *Scheme $A_{2}$ is k-server t-private b-Byzantine-resistant PIR over $F_{q}$ with file size $\left(r-t-2b\right)\mathrm{lcm}\left(k_{2}-t-2b,\ldots ,k_{d}-t-2b\right)\log\left(q\right)$ bits and retrieval threshold $r=k_{1}$, which achieves the asymptotic capacity (3) for any given r so that $t<r-2b<k_{2}-2b<\ldots <k_{d}-2b=k-2b\leq k\leq q$.*

Let us start with numerical comparison. Let us consider a PIR scheme with 27 servers deployed over $F_{32}$ with t=1 and b=1, allowing recovery from {11,19,27} servers. Each file consists of s=8 components. As a result, each file in scheme $\Pi_{2}$ is eight elements of $F_{32^{6}}$ or 240 bits, while each file in scheme $A_{2}$ is eight vectors of 48 elements of $F_{32}$ or 1920 bits. Let us move to the retrieval process in the case of 27 involved servers. In the case of $\Pi_{2}$, we need to download 27 traces of ${\mathrm{Tr}}_{F_{32^{6}}/F_{32^{2}}}$ that are elements of $F_{32^{2}}$ or 270 bits and decode one codeword of generalized Reed–Solomon code over $F_{32^{2}}$. In the case of $A_{2}$, we need to download eight vectors of length 54 over $F_{32}$ or 2160 bits and decode eight codewords of generalized Reed–Solomon code over $F_{32}$. The same holds for other parameter values. For the convenience of the reader, we provide the values of the field size, file size, and download cost for the case of $k_{d}$ participating servers in each scheme in Table 2. We denote $L_{0}=lcm\left(k_{2}-t,\ldots ,k_{d}-t\right)$, $L_{b}=lcm\left(k_{2}-t-2b,\ldots ,k_{d}-t-2b\right)$, and measure all parameters in bits.

Although our schemes ($\Pi_{1}$ and $\Pi_{2}$) are deployed over extended fields, we operate on single field elements and perform operations over the extended field. These operations can therefore be optimized [13]. In contrast, competing schemes ($A_{1}$ and $A_{2}$) are deployed over base-field operands with vectors whose lengths are larger than the extension degrees in our case. This makes them slower and limits potential optimization approaches. Another advantage of our scheme is that, in Byzantine recovery, we decode only one generalized Reed–Solomon code in an intermediate field, which does not significantly increase complexity over the base field. In comparison, $A_{2}$ must decode several generalized Reed–Solomon codes, making it more computationally heavy than our approach.

## 6. Conclusions

We considered the problem of designing a Private Information Retrieval scheme resistant to the adversarial behavior of servers. Our focus was on minimizing download costs, and we proposed a capacity-achieving scheme with a small file size for an asymptotically large number of files of fixed size. To address the possible presence of slow or unresponsive servers, whose number is not known or may change over time, we proposed schemes in which the number of servers participating in retrieval may vary, extending the non-universal results presented in the conference. Additionally, we formally proved the optimality of the file size in the proposed schemes that significantly affect implementation complexity. Extending the proposed framework to a finite number of files, as well as considering other Byzantine PIR models, including those with a limited-knowledge adversary or vanishing probability of error, remains an interesting open problem.

## Figures and Tables

**Table 1:** Notation table.

Symbol	Description
*k*	Number of servers
*t*	Number of colluding servers
*b*	Number of Byzantine servers
*r*	Minimum number of servers required for retrieval (retrieval threshold)
*n*	Number of files in the database
*s*	Sub-packetization
x	Database
$F_{x}$	Multivariate polynomial representing the database x
$q_{i}$	Query to server *i*
$a_{i}$	Answer (response to the query) from server *i*
$x_{i}$	Element *i* of vector x
*T*	Transpose operations
[n]	Set of integers {1,…,n} for n>0
$N^{*}$	Set of all positive integers
$F_{q^{e}}$	Extension field with $q^{e}$ elements
$F_{q}$	Base field with *q* elements
${\mathrm{Tr}}_{F_{q^{e}}/F_{q^{\lambda }}}$	Trace-mapping function from $F_{q^{e}}$ to its subfield $F_{q^{\lambda }}$
$F_{q^{e}}\left[\xi \right]$	Ring of polynomials over $F_{q^{e}}$

**Table 2:** Parameters of PIR schemes with optimal download rate for asymptotically large numbers of files.

	$\Pi_{1}$	$\Pi_{2}$	$A_{1}$	$A_{2}$
File size	$L_{0}\log\left(q\right)$	$L_{b}\log\left(q\right)$	$\left(r-t\right)L_{0}\log\left(q\right)$	$\left(r-t-2b\right)L_{b}\log\left(q\right)$
Field of operations	$F_{q^{e}}$, where $e=\frac{L_{0}}{r-t}$	$F_{q^{e}}$, where $e=\frac{L_{b}}{r-t-2b}$	$F_{q}$	$F_{q}$
Download cost	$\frac{k_{d}}{k_{d}-t}L_{0}\log\left(q\right)$	$\frac{k_{d}}{k_{d}-t-2b}L_{b}\log\left(q\right)$	$\frac{k_{d}}{k_{d}-t}L_{0}\left(r-t\right)\log\left(q\right)$	$\frac{k_{d}}{k_{d}-t-2b}L_{b}\left(r-t-2b\right)\log\left(q\right)$
Byzantine resistance	0	b≠0	0	b≠0

## Data Availability

The original contributions presented in this study are included in the article. Further inquiries can be directed to the corresponding author.
